# Supplementary material for: Characteristics and Relationships between Total Polyphenol and Flavonoid Contents, Antioxidant Capacities, and the Content of Caffeine, Gallic Acid, and Major Catechins in Wild/Ancient and Cultivated Teas in Vietnam
Source: Molecules. 2023 Apr 14;28(8):3470. doi: 10.3390/molecules28083470 (PMC10142074; doi:10.3390/molecules28083470)
Supplement: Supplementary file 1 [file molecules-28-03470-s001.zip › molecules-2229546-supplementary.pdf]

molecules

MDPI

Table S1. Tea sample coding and their origins.

Sample code	Tea type	Geographic origin	GPS coordinates	Production date	Tea leaf grading
DL-G-01	Green tea	Da Lat city (Lam Dong Province) Central highlands South of Vietnam	11°52'39.0"N 108°33'40.5" E 11.877487, 108.561258	12/2020	1 bud + 2 leaves (old tea tree)
DL-G-02				06/2022	1 bud + 2 leaves (old tea tree)
DL-G-03				10/2020	1 bud + 2-3 leaves
DL-G-04				06/2022	1 bud + 2-3 leaves
DL-O-01	Oolong tea			06/2021	1 bud + 2-3 leaves
DL-O-02				12/2020	1 bud + 2-3 leaves
DL-O-03				10/2021	1 bud + 2-3 leaves
DL-O-04				06/2022	1 bud + 2-3 leaves
DL-O-06				06/2021	1 bud + 2-3 leaves
DL-O-08				06/2022	1 bud + 2-3 leaves
LH-O-01	Oolong tea	Lam Ha district (Lam Dong Province) Central highlands South of Vietnam	11°47'21.5"N 108°09'49.9" E 11.789306, 108.163849	10/2020	1 bud + 2-3 leaves
LH-O-02				04/2021	1 bud + 2-3 leaves
LH-O-03				06/2021	1 bud + 2-3 leaves
LH-O-05				12/2020	1 bud + 2-3 leaves
LH-O-06				04/2021	1 bud + 2-3 leaves
LH-O-07				06/2021	1 bud + 2-3 leaves
LH-O-08				09/2021	1 bud + 2-3 leaves
SG-G-01	Green tea	Suoi Giang commune (Yen Bai Province) North of Vietnam	21°37'33.7"N 104°36'53.3" E 21.626020, 104.614804	11/2020	1 bud
SG-G-02				06/2021	1 bud
SG-G-03				07/2022	1 bud
SG-G-04				11/2020	1 bud + 1 leaf
SG-G-05				04/2021	1 bud + 1 leaf
SG-G-06				07/2022	1 bud + 1 leaf
SG-G-07				11/2020	1 bud + 2 leaves
SG-P-01	Raw Pu'erh tea			09/2020	1 bud + 1 leaf
SG-P-02				10/2020	1 bud + 1 leaf
SG-B-01	Black tea			10/2020	1 bud + 2 leaves
SG-B-02				06/2021	1 bud + 2 leaves
SG-B-03				04/2022	1 bud + 2 leaves

Table S2. The mass spectrometry conditions for analysing caffeine, gallic acid and catechins by UPLC-MS/MS

**Compound**	**ES mode**	**Precursor ion (m/z)**	**Product ion (m/z)**	**Cone (V)**	**Collison (V)**
GA	(−)	168.95	78.95 (C)	35.0	20.0
		168.95	125.00 (Q)	35.0	15.0
CFI	(+)	195.02	110.00 (C)	40.0	25.0
		195.02	138.02 (Q)	40.0	20.0
EC	(−)	288.95	203.00 (C)	40.0	20.0
		288.95	245.00 (Q)	40.0	15.0
C	(−)	289.00	203.00 (C)	40.0	20.0
		289.00	245.05 (Q)	40.0	15.0
EGC	(−)	305.00	125.00 (Q)	40.0	25.0
		305.00	179.00 (C)	40.0	15.0
ECG	(−)	440.95	168.95 (C)	35.0	20.0
		440.95	289.05 (Q)	35.0	20.0
EGCG	(−)	457.00	125.00 (C)	35.0	40.0
		457.00	168.95 (Q)	35.0	17.0

C: confirmation m/z; Q: quantitation m/z

Table S3. Parameters of regression equations.

Criteria	Results
TPCs	Working range: 10-70 (mg GAE L^{-1})<br>$y = 0.0117x + 0.0221$, $R^2 = 0.99946$, Bias: 0.13-4.96%
TFCs	Working range: 50-700 (mg QE L^{-1})<br>$y = 0.0013x + 0.1454$, $R^2 = 0.9980$, Bias: 0.09-8.48%
DPPH	Working range: 100-700 (μmol TE L^{-1})<br>$y = 0.0011x + 0.0174$, $R^2 = 0.9991$, Bias: 0.06-9.49%
ABTS	Working range: 100-700 (μmol TE L^{-1})<br>$y = 0.0009x – 0.0111$, $R^2 = 0.9985$, Bias: 0.2-10.45%
FRAP	Working range: 100-700 (μmol TE L^{-1})<br>$y = 0.0011x + 0.0113$, $R^2 = 0.9982$, Bias: 0.01-14.2%
CUPRAC	Working range: 100-700 (μmol TE L^{-1})<br>$y = 0.0011x + 0.0127$, $R^2 = 0.9979$, Bias: 0.42-9.55%
Caffeine (CFI)	Working range: 1-40 (mg L^{-1})<br>$y = 1401x – 287300$, $R^2 = 0.9996$
Gallic acid (GA)	Working range: 5-500 (μg L^{-1})<br>$y = 382.5x – 379$, $R^2 = 0.9991$
Catechin (C)	Working range: 0.5-20 (mg L^{-1})<br>$y = 94.09x – 6778$, $R^2 = 0.9990$
Epicatechin (EC)	Working range: 0.5-30 (mg L^{-1})<br>$y = 64.81x – 6810$, $R^2 = 0.9995$
Epicatechin gallate (ECG)	Working range: 0.5-30 (mg L^{-1})<br>$y = 185.1x – 23860$, $R^2 = 0.9993$
Epigallocatechin (EGC)	Working range: 0.5-20 (mg L^{-1})<br>$y = 56.87x – 2303$, $R^2 = 0.9990$
Epigallocatechin gallate (EGCG)	Working range: 5-50 (mg L^{-1})<br>$y = 199.6x – 23540$, $R^2 = 0.9997$

Table S4. LODs and LOQs.

	LOD	LOQ		LOD (μg L^{-1})	LOQ (μg L^{-1})
TPCs	0.81	2.7	CFI	0.83	2.8
TFCs	5.1	15.6	GA	0.37	1.2
DPPH	16.3	49.5	C	0.48	1.6
ABTS	12.3	37.3	EC	0.58	1.9
FRAP	9.9	30.1	EGC	0.8	2.7
CUPRAC	11.8	35.7	ECG	0.72	2.4
			EGCG	0.86	2.9

Table S5. Repeatability and reproducibility.

	**RSD$_r$ (%)**	**RSD$_R$ (%)**
TPCs	0.79	1.23
TFCs	1.14	1.31
DPPH	1.5	1.63
ABTS	1.37	1.48
FRAP	1.03	1.26
CUPRAC	1.52	1.55
CFI	1.4-1.7	1.4-1.7
GA	1.1-1.6	1.2-1.7
C	1.4-2.0	1.4-2.0
EC	0.90-1.6	0.96-1.6
EGC	0.39-1.7	0.39-1.7
ECG	0.69-1.9	0.69.1.9
EGCG	1.1-1.7	1.1-1.7

Table S6. Recovery test.

	**Spiked**		
	**0.5Cx**	**Cx**	**1.5Cx**
TPCs	98.9	99.4	98.8
TFCs	98.2-101	99.1-99.8	98.5-101
DPPH	98.0-99.5	98.6-99.4	99.3-100
ABTS	99.3-99.6	98.7-99.8	99.7-101
FRAP	98.7-98.9	99.6-100	99.1-99.9
CUPRAC	98.0-100	98.5-99.5	98.9-99.6
CFI		100	
GA		93.8	
C		94.3	
EC		99.9	
EGC		98.8	
ECG		98	
EGCG		97.1	
